# Cannabis in Medicine: An Evidence-Based Approach

**DOI:** 10.5195/jmla.2021.1211

**Published:** 2021-04-01

**Authors:** Melanie J. Norton

**Affiliations:** 1 melanie.norton@yale.edu, Harvey Cushing/John Hay Whitney Medical Library, Yale University, New Haven, CT

Did you know that, after alcohol and tobacco, cannabis is the most-used drug in the United States? We learn this on page 41 of *Cannabis in Medicine: An Evidence-Based Approach*, edited by Kenneth Finn, MD, a text that provides an overview of studies on the medicinal use of cannabis as well as areas that still need scientific study. Dr. Finn received his medical degree from the University of Texas Health Science Center at Houston in 1990 and currently practices in Colorado Springs, where he specializes in pain medicine, physical medicine, and rehabilitation. For his book, he has gathered several highly regarded medical doctors from all over the world to contribute to his publication.

The book is divided into two sections. Part I, comprising three chapters, focuses on the basic science of marijuana. A list of abbreviations at the beginning of chapter 1 proves useful for the non-medical reader of this dense publication. Besides providing the chemical compounds of cannabis, part I discusses the social, political and physiological background of cannabis use. The political and social climate has changed within the United States, so, too, has the use of marijuana. With its use becoming more acceptable, more states are decriminalizing cannabis, both for recreational and medicinal purposes. Thus, it is more important now than ever to look at the evidence-based medical research as to how marijuana use affects and interacts with a person's health.

Part II focuses on the need for more studies to determine how cannabis, whether used for recreational purposes or medical alternatives, interacts with other prescribed medications. Included here are evidence-based studies on the effects of cannabis as it relates to several health-related issues, such as neuropsychiatry, adolescence development, pain medicine, and the cardiovascular system. Part II also discusses the economics of marijuana and especially the positive impact many states have felt from high taxes placed on sales of recreational marijuana. Like a cigarette tax, the tax on marijuana creates a complex dilemma, and chapter 18 deals with this: “Public health messaging must resonate with shifting public attitudes to marijuana use, and public health policies must be negotiated with consumers as well as economic operators” (p. 473). This chapter also sums up the premise of the book. The ship has sailed as far as decriminalizing marijuana is concerned, and it will take time for the research, not only on public health but on all aspects of health, to catch up.

The illustrations and charts, some in color, provide visual support of the research. *Cannabis in Medicine: An Evidence-Based Approach* is a specialized resource appropriate for medical or hospital libraries to have on hand for clinicians and policymakers.

